# How Low-Frequency Neural Activity Structures Language in Time

**DOI:** 10.1162/NOL.a.249

**Published:** 2026-05-05

**Authors:** Lena Henke, Burkhard Maess, Lars Meyer

**Affiliations:** Max Planck Research Group Language Cycles, Max Planck Institute for Human Cognitive and Brain Sciences, Leipzig, Germany; Methods and Development Group Brain Networks, Max Planck Institute for Human Cognitive and Brain Sciences, Leipzig, Germany; Clinic for Phoniatrics and Pediatric Audiology, University Hospital Münster, Münster, Germany

**Keywords:** chunking, closure positive shift, delta-band oscillations, phase-locking, temporal constraint

## Abstract

The integration of sensory information in humans may be confined to a time window of 2–3 s. In language, this time window constrains the grouping of words into multi-word chunks, required for comprehension. Chunk boundaries are known to elicit a characteristic event-related brain potential, the Closure Positive Shift (CPS). The likelihood of a CPS increases with the duration of the chunk. In the frequency-domain, boundaries have been associated with neural oscillations in the delta band (<4 Hz). Here, we assessed whether the pace for chunking might be imposed by electrophysiological processing cycles of the brain with phase-locking of such activity underlying the CPS. We recorded participants’ magnetoencephalogram while they listened to globally ambiguous sentences allowing for two alternative ways of chunking. Chunking was not externally imposed, but the temporal limits of integration windows influenced chunk termination. Phase-locking of narrow-band low-frequency neural activity (i.e., <4 Hz) at the boundaries of multi-word chunks increased with sentence duration, and covaried with event-related fields. Behavioral data further indicate subtle interindividual differences in the duration of the integration time window. Source localization revealed neural generators in bilateral posterior temporal and right anterior regions. The brain appears to project duration-limited integration windows onto the incoming auditory speech signal, thus structuring language comprehension in time.

## INTRODUCTION

Language comprehension requires words to be linked into multi-word chunks. Chunking divides the input into groups of words, independent of their internal structure (Abney, 1991). This does not only necessitate the processing of current stimuli, but also stimuli that appeared seconds ago. Due to deterioration of working memory contents, multi-word chunks are limited in time (Christiansen & Chater, 2016). Longer durations of chunks increase the likelihood of the insertion of a chunk boundary (Hirose, 2003; Hwang & Schafer, 2009; Webman-Shafran & Fodor, 2016). This is indicated by a characteristic event-related brain potential (ERP) in the electroencephalogram (EEG) at chunk boundaries, the *Closure Positive Shift* (CPS; Steinhauer et al., 1999). CPS amplitude increases with chunk duration (Hwang & Steinhauer, 2011) and is elicited independently of concurrent or absent physical markings of chunk boundaries (Drury et al., 2016; Itzhak et al., 2010; Roll et al., 2012; Steinhauer & Friederici, 2001; for review, see Bögels et al., 2011). The CPS can index temporal limitations of chunking, as it exhibits an intrinsic duration preference: occurrence likelihood increases when chunks last 2.7 s (Roll et al., 2012).

Given its intrinsic period, it was previously hypothesized that the CPS has a periodic electrophysiological substrate (Henke & Meyer, 2021; Inbar et al., 2023; Meyer et al., 2017), where an increase in evoked amplitude might result from an increase in phase-locking of underlying neural oscillations across trials (Sauseng et al., 2007). Specifically, it could be the time-domain equivalent of phase-locked narrow-band activity at delta-band frequency (<4 Hz). The CPS and delta-band activity are modulated by similar experimental manipulations: Both are involved in the active insertion of chunk boundaries (Ding et al., 2016; Gilbert et al., 2015; Meyer et al., 2017) and respond to (implicit) prosody (Bourguignon et al., 2013; Glushko et al., 2022; Steinhauer et al., 1999). Both have been suggested to serve as a pacemaker for temporal grouping (Boucher et al., 2019; Henke & Meyer, 2021; Meyer et al., 2020a), such as the integration of individual words into larger multi-word chunks. Moreover, just like the CPS, delta-band phase predicts the chunking of words irrespective of prosody (Chalas et al., 2024; Meyer et al., 2017) and seems to enforce the insertion of chunk boundaries after 2.7 s (Henke & Meyer, 2021). In analogy to this endogenous involvement in the chunking of words, auditory digit retrieval remains high only as long as the duration of acoustically marked multi-digit chunks fit the period of delta-band oscillations (Ghitza, 2017; Rimmele et al., 2021).

**Figure 1.**
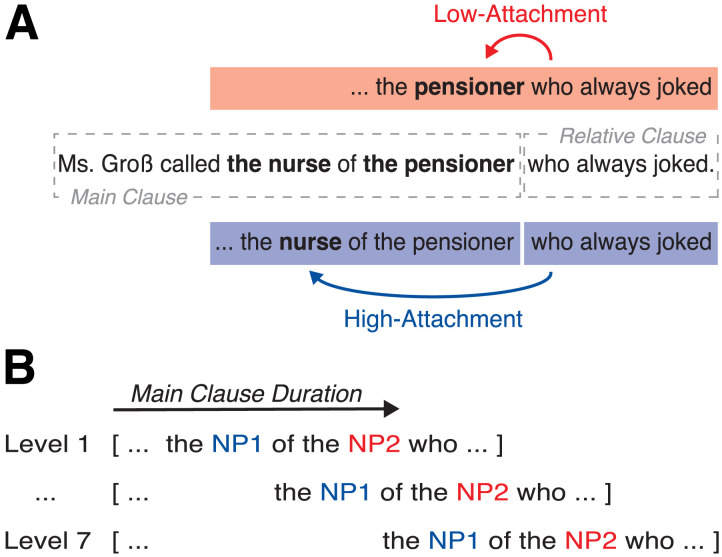
Materials and experimental manipulation. (A) An example of a relative clause attachment ambiguity, where the relative clause can be combined with either of two different noun phases. When the relative clause is combined with the first noun phrase (blue) this is called high attachment in psycholinguistics. When it is linked to the second noun phrase (red), this is called low attachment. (B) The experiment manipulated the duration of the main clause in seven levels in order to parametrically increase the likelihood of the insertion of a chunk boundary at the end of the main clause; chunking should influence sentence comprehension (i.e., relative clause attachment).

To assess the potential functional and neural equivalence of the CPS and delta-band oscillations, the current auditory magnetoencephalography (MEG) study manipulated the duration of multi-word chunks up to a potential chunking point, at which analyses were performed. We employed sentences containing ambiguous relative clauses (RCs; e.g., *Ms. Gross called the nurse of the pensioner who always joked.*), where the RC (i.e., *who always joked*) can be combined with either of two preceding noun phrases (i.e., NP1 *the nurse* or NP2 *the pensioner*; Figure 1A), leading to different interpretations. The interpretation chosen by the listener reflects chunking: Insertion of a chunk boundary after NP2 means that the RC is combined with NP1 (Carlson et al., 2001; Clifton et al., 2002; Frazier et al., 2006), whereas combination of the RC with NP2 would go without a boundary after NP2. To assess phase-locking and event-related fields (ERFs) as a function of sentence duration, we created seven levels ranging from ∼2–4 s in duration. This was achieved by manipulating the length of words (i.e., increasing the number of syllables; Figure 1B). We hypothesized that the likelihood of inserting a chunk boundary after NP2 (i.e., *the pensioner* in the above example) increases with sentence duration. Given prior evidence on inter-individual differences in chunking based on working memory capacity (Roll et al., 2012; Schremm et al., 2015; Swets et al., 2007), we additionally reasoned that chunking decisions are influenced by individuals’ working memory span: Individuals with a high span might be able to chunk information over longer sentences. This could reflect an endogenous limit on chunk duration—set by an intrinsic neural period.

## MATERIALS AND METHODS

### Participants

Forty German native speakers participated in the experiment (21 female; mean age = 27 years, standard deviation (*SD*) = 5 years). Participants were right-handed (Oldfield, 1971; mean lateralization quotient = 90, *SD* = 12) and reported no history of neurological or hearing disorder. The study was approved by the ethics committee of the University of Leipzig according to the Declaration of Helsinki. Informed consent was given prior to participation.

### Materials

We employed globally ambiguous relative clauses (RCs; e.g., *Ms. Gross called the nurse of the pensioner who always joked.*). Such sentences are termed ambiguous because the RC (i.e., *who always joked*) can modify (i.e., be linked or attached to) either of two preceding noun phrases (i.e., NP1 *the nurse* or NP2 *the pensioner*; see Figure 1A). The link to NP1 is called high attachment, whereas the link to NP2 is called low attachment. Generally, attachment was suggested to reflect the chunking decision of a listener: insertion of a chunk boundary after NP2 leads to NP1 attachment (Carlson et al., 2001; Clifton et al., 2002; Frazier et al., 2006). In contrast, when NP2 and the relative clause fall into a single chunk, this leads to NP2 attachment. Prior work has found that the tendency for NP1 attachment increases for long RCs (e.g., *who tragically died of a stroke* as compared to *who died*), thus favoring moderate chunk durations (Hemforth et al., 2015; Jun & Koike, 2008). In addition, individuals with low working memory capacity end chunks earlier (i.e., insert a boundary) than individuals with high working memory capacity (James et al., 2018; Swets et al., 2007; although, see Traxler, 2007, for opposite results), leading to more NP1 attachment.

To target specifically the proposed time constraint of 2.7 s, we parametrically manipulated the duration of sentences containing ambiguous relative clauses by increasing the number of syllables within words while keeping the number of words constant. Thus, the syntactic structure was unchanged (Figure 1B). We created seven levels with increasing main clause duration ranging from around 2 s (level one) to 4 s (level seven). For details on all levels, see Table 1. To account for attachment biases, possible attachment sites (i.e., NP1 and NP2) contained the same number of syllables (Colonna, 2001; Wijnen, 2004) and were either both female or male nouns to account for gender markings in German. In order to avoid additional confounds, the length of the RCs was kept constant (i.e., three words, five syllables; Hemforth et al., 2015; Pynte & Colonna, 2000). For the shortest and longest levels, we created 25 sentences. For all other levels, we initially created 50 sentences. For the final randomization, each participant listened to 25 trials within each level, resulting in a total of 175 experimental sentences across conditions. More details on the construction of the sentences can be found in the Supplementary Materials (Supporting Information can be found at https://doi.org/10.1162/NOL.a.249). Sentences were synthesized using WaveNet voices (van den Oord et al., 2016) provided by the Google Cloud Text-to-Speech API. Synthesized speech was chosen over recordings of a human speaker to have a consistent speech signal without requiring additional audio manipulations.

**Table 1.** Examples and descriptives of the seven levels

**Level**	**Example sentence** (main clause in bold)	**Main clause length**	
		**in syllables**	**in seconds: mean (*SD*)**
1	**Frau Groβ rief den Pfleger des Rentners** der durchweg scherzte.	9	2.09 (0.14)
	***Ms Gross called the nurse of the pensioner** who always joked.*		
2	**Frau Lindner vernahm den Partner des Räubers** der vorhin tobte.	11	2.35 (0.14)
	***Ms Lindner heard the partner of the robber** who had been raving earlier.*		
3	**Herr Pfeiffer stützte den Großvater des Besuchers** der ständig fluchte.	13	2.66 (0.14)
	***Mr Pfeiffer supported the grandfather of the visitor** who was constantly swearing.*		
4	**Herr Jacobi zeichnete den Bergführer des Wanderers** der stark nuschelte.	15	2.94 (0.12)
	***Mr Jacobi drew the mountain guide of the hiker** who mumbled heavily.*		
5	**Herr Engelbrecht bedrohte den Fotografen des Galeristen** der montags joggte.	17	3.23 (0.17)
	***Mr Engelbrecht threatened the photographer of the gallery owner** who goes running on Mondays.*		
6	**Frau Kaltenhauser ermutigte den Unterstützer des Finalisten** der langsam aufgab.	19	3.47 (0.16)
	***Ms Kaltenhauser encouraged the supporter of the finalist** who slowly gave up.*		
7	**Herr Hintermeier kontaktierte den Repräsentanten des Außenministers** der jüngst verreiste.	21	3.78 (0.16)
	***Mr Hintermeier contacted the representative of the Minister of Foreign Affairs** who recently travelled.*		

To exclude the possibility that potentially observed differences in attachment are driven by systematic differences in acoustic boundary strength between levels, we determined boundary strength in each sentence by using the Wavelet Prosody Toolkit (Suni, 2017; Suni et al., 2017). This algorithm estimates the strength of word boundaries based on the fundamental frequency, intensity, and duration using time-aligned word annotations. We correlated the boundary strength at the potential chunking position (i.e., the offset of NP2) with the main clause duration (i.e., the sentence duration up to this point) across all sentences. Kendall’s rank correlation indicated no systematic relationship between duration and boundary strength (r_*τ*_ = 0.03, *p* = 0.39), indicating that this potential confound was not an issue.

### *N*-Back Task

Previous research has indicated the relevance of working memory capacity for chunking. For instance, individuals with high working memory capacity appear to integrate (more) information over longer temporal intervals (James et al., 2018; Swets et al., 2007; although, see Traxler, 2007, for opposite results). Additionally, there is a relation between the amplitude of the CPS and individuals’ working memory capacity (Roll et al., 2012; Schremm et al., 2015). Therefore, in the present study, we assessed working memory capacity using an *n*-back task (Braver et al., 1997). Eight capital consonant letters (C, G, H, K, P, Q, T, W; Jaeggi et al., 2010) served as stimuli. Each memory load (2–, 3–, and 4-back) was presented for three consecutive blocks. Each block contained 20 + *n* items, including six targets. One block of 1-back served as practice. Letters were presented for 500 ms with an inter-stimulus interval of 2,500 ms. As a result, participants had 3 s to respond via a button press if the letter matched the one *n* trials ago. Participant’s score was calculated as the proportion of hits minus false alarms. Three participants did not complete all blocks, indicating too high difficulty; therefore, their scores were calculated assuming that all remaining targets would have been missed. For further analyses, we divided participants into groups with high and low working memory span based on a median split.

### Procedure

At the beginning of the session, each participant’s hearing threshold was tested for each ear to determine an individual audio level for later stimulus presentation (45 dB sensation level; i.e., above individual’s hearing threshold). To that end, a test sentence was presented with continuously changing intensity while participants needed to indicate via a button press when they could no longer hear, or started to hear the sound. Audio was presented via in-ear headphones.

During MEG recording, participants were seated in an electromagnetically shielded room. First, we recorded participants’ resting-state MEG. Here, participants sat for 5 min with their eyes open and then with their eyes closed without any stimulation (Beese et al., 2017). These data are not analyzed here. During the experimental session, participants listened to sentences of different lengths presented using Presentation (Neurobehavioral Systems, Inc., Albany, US). The main experiment was conducted in six recording blocks, with 30 experimental trials in the first five blocks and 25 in the last one, resulting in 175 trials in total. Each target sentence was interleaved with a filler sentence unrelated to the experimental manipulation (Friederici et al., 2006; Meyer et al., 2012) to avoid any kind of priming based on the length of the previous sentence (Lamekina & Meyer, 2023; Lamekina et al., 2024). Participants could take a break in between blocks. Each trial started with a green fixation cross (1,500 ms), which transitioned to red (500 ms) for stimulus playback. A jitter interval (range: 100–600 ms) preceded playback to avoid coupling between sensory modalities (Lakatos et al., 2008; Power et al., 2012). That is, the visual cue might lead to an amplification of the neural response in auditory cortices, possibly mediated by a phase reset. After playback, the fixation cross transitioned back in color. Subsequently, the comprehension question—targeted at revealing the attachment (i.e., for the example above *Who joked always?*)—was shown with the two answer options underneath (i.e., *the nurse*, *the pensioner*). The order of answer options was counterbalanced within participants and conditions. Participants were instructed to answer as quickly and intuitively as possible. The response was given via a button press and could be made within 3 s until timeout. The same procedure also applied to filler sentences. Each filler was followed by a comprehension question with two persons as answer options; yet, answers were unambiguous. At the beginning of the experiment, participants performed seven additional trials to familiarize themselves with the procedure. After the MEG recording, participants took a short break and then performed the *n*-back task outside of the shielded room. An entire session took approximately 3.5 h.

### Data Acquisition

We recorded MEG using a 306-channel Elekta Neuromag system (Elekta Neuromag Oy, Helsinki, Finland) in an electromagnetically shielded room (each channel comprised of one magnetometer and two orthogonal planar gradiometers). Magnetic signals were recorded at a sampling rate of 1000 Hz within a frequency band from DC to 330 Hz. Five Head Position Indicator (HPI) coils attached to the scalp were used to continuously monitor head movements. Behavioral responses were recorded via a button box.

### Electrophysiological Analysis

The MEG data were first spatially filtered using Elekta Neuromag MaxFilter utilizing spherical functions up to the eleventh order for the head field model and up to the second order for the environmental field model. As some participants showed a head displacement larger than 5 mm (mean head displacement = 4.1 mm, *SD* = 2.7 mm, range = 0.3–19.4 mm), we applied the filter with the temporal Signal Space Separation method (tSSS; Taulu & Simola, 2006). The temporal correction was computed on 10-s time windows; correlations higher than 0.98 between inside and outside field components were projected out. The filter was used to suppress environmental noise, interpolate manually identified bad or broken channels (mean number of channels = 7, *SD* = 1.9), and project all blocks to the initial head position of the first block (Taulu et al., 2004). The same channels were interpolated across all blocks.

Further data analysis was conducted in MATLAB (The MathWorks, Inc.) using custom code and functions from Fieldtrip (Oostenveld et al., 2011). Analysis was performed based on magnetometers only, as the information across channel types (i.e., magnetometers and gradiometers) is equalized after SSS, and their results are highly correlated (Garcés et al., 2017). First, the continuous signal was 0.2-Hz high-pass filtered and 30-Hz low-pass filtered with a two-pass Butterworth *infinite-impulse-response* (*IIR*) filter of 5^th^ and 14^th^ order, respectively. Then, data were segmented into epochs of 6 s, extending from ±3 s around the offset of the main clause. In order to remove SQUID jump and muscle artifacts, epochs were rejected when z-scored values of the 9^th^-order median-filtered signal exceeded 60, or the signal exceeded ±5 pT in any channel. Automatic artifact rejection led to an average removal of 0.2 trials (*SD* = 0.5) per participant. Finally, we applied independent component analysis (ICA; Makeig et al., 1995) to remove eye-blink and heartbeat artifacts. ICA was computed after PCA dimension reduction to 20 components. On average, 3 (*SD* = 0.85) components were removed per participant. Finally, the remaining trials were manually inspected for artifacts (mean number of removed trials = 11, *SD* = 8). In the end, data was down-sampled to 100 Hz.

### Sensor-Level Analysis

First, we tested if phase-locking of low-frequency oscillatory activity is related to clause duration. To that end, we low-pass filtered the data at 4 Hz with a two-pass Butterworth *IIR* filter of 8^th^ order. We then derived the analytic phase of the narrow-band signal through a Hilbert transform and, finally, computed the inter-trial phase coherence (ITPC) across trials within each level. For statistical analysis, we performed a cluster-based linear regression analysis across levels within the time window from 0–1 s after the main clause offset (Maris & Oostenveld, 2007); two-sided, *α* = 0.05, 10,000 permutations, ≥3 channels minimum cluster size).

Secondly, we tested if the amplitude of the ERF changes with main clause duration. That is, when sentences get longer, does the likelihood of inserting a boundary increase? On that end, we computed the ERF for each level around the main clause offset. The cluster-based regression analysis across levels was the same as for the ITPC (0–1 s after the main clause offset; two-sided, *α* = 0.05, 10,000 permutations, ≥3 channels minimum cluster size).

Lastly, we were interested in the relationship between the ERF and the oscillatory activity. In order to investigate whether the evoked response corresponds to phase-locked delta-band activity, we analyzed the relation between the ERF amplitude and the ITPC. For each level, we extracted the ITPC at the magnetometers and time points revealed by the observed clusters (see Results). Likewise, we masked the ERF based on the observed clusters of the ITPC analysis; that is, we extracted the amplitude from the magnetometer and time points of the ITPC cluster. For each timepoint and magnetometer, we fitted a linear mixed-effects model for the ERF amplitude with ITPC as fixed effect and random intercepts for participants in R (R Core Team, 2021). *P* values were calculated based on model comparison against a baseline model without the factor of interest (i.e., ITPC). Within each cluster, the results were Bonferroni-corrected to account for multiple comparisons across magnetometers and time points.

### Source-Level Analysis

For localization of the neural source underlying the phase-locking, we applied anatomically constrained source localization. First, we used Freesurfer (https://surfer.nmr.mgh.harvard.edu/) to reconstruct the inner skull surface and segment the cortical surface of individual structural T1-weighted MRI images. The cortical surface was divided into a grid of source positions consisting of 10,242 vertices per hemisphere with MNE. Then, the forward solution was computed based on the individual MRI images: We constructed individual, single shell volume conductors as boundary element models (BEMs). For the inner-skull surface triangulation comprising 2,562 vertices each, we used the watershed algorithm. Individual head models were created based on these volume conductors using a single compartment model (Nolte, 2003). Next, the MEG data were co-registered with individuals’ MRI images via alignment of the fiducial (left and right (peri)auricular, nasion) and a large number of head surface points digitized with a Polhemus FASTRAK 3D digitizer. We used a semi-automated procedure based on a combination of the Iterative-Closest-Point (ICP; Besl & McKay, 1992) algorithm implemented in MNE and manual fine-tuning.

All further source-space analysis was conducted in MATLAB using FieldTrip functions. For group-level statistics, we warped the positions of the MNI template grid with a 5 mm resolution included in FieldTrip to the individual MRI. Thus, source reconstruction was done in individual space, while group-level statistics were performed in MNI space. Finally, lead fields were calculated separately for each subject and block based on previously constructed head models, warped source spaces, and the MEG sensor positions.

For calculating ITPC in source space, we continued with the down-sampled and 4-Hz low-pass filtered data and followed the Hilbert beamformer pipeline (Jensen et al., 2019, 2023; Westner, 2017; Westner & Dalal, 2019). First, we calculated the sensor covariance matrices across all conditions extending over the time window of the observed ITPC clusters in the sensor-level analysis ±1 second (see Results; −0.85–1.38 s). Then, we used a linear constrained minimum variance (LCMV) beamformer (Van Veen et al., 1997) with unit-noise-gain against depth bias (Westner et al., 2022) to compute the source activity on the cortical surface. The common filter was computed with a fixed orientation on the axis of most variance, and the regularization parameter *λ* was set to 5%. Afterwards, the single-trial data were Hilbert-transformed, and the filter was applied to the analytic signal, resulting in a source reconstruction of analytic magnitude and phase for each trial and each node in the lead field. Finally, we calculated the ITPC for each node and condition across blocks.

In order to interpret the source-level results in terms of neuroanatomical structure and function, we parcellated the template brain into regions of the HCPMMP1 atlas (Glasser et al., 2016). For statistical analysis, we then masked the nodes so that only those that fell into any atlas-defined cortical region remained (4,672 nodes). Statistical analysis was performed using a cluster-based regression analysis across levels averaged across the time window of both clusters (0.15–0.38 s after the main clause offset; one-sided, *α* = 0.05, 10,000 permutations). Neighboring nodes were defined in all dimensions, leading to a maximum of 26 neighbors; a node has fewer neighbors when it is at an outer edge position. Note that we here performed one-sided testing based on the sensor-level results (see Results), which were already known at this point.

### Behavioral Analysis

After each sentence, participants indicated via a button press their attachment site. We first investigated whether the behavioral response changed according to the duration manipulation. On that end, we fitted a generalized linear mixed effects model with binomial link function on the single trial response with the ordered factor level as fixed effect and random intercepts for participants. Time-outs were removed before the analysis (mean number of removed trials = 1, *SD* = 2.6).

Additionally, we were interested in a relationship between the behavioral preference and the neural results. Here, we removed responses to trials that were excluded from the MEG analysis and calculated the percentage of low attachment for each level. Note the number of behavioral answers might not always be exactly the same as in the ITPC analysis due to additional timeouts in the behavioral responses. We fitted a linear mixed-effects model on the attachment using the R package *buildmer* (Voeten, 2025) to determine the maximal model based on the automatic stepwise selection applied with forward effect selection. The initial model included ITPC (numeric predictor) and *n*-back group (sum contrast-coded) as well as their interaction term as fixed effects, and random intercepts for participants. ITPC was extracted from the magnetometer and timepoint that showed the strongest relationship with the amplitude across the broadband data (see Results).

## RESULTS

### Delta-Band Phase-Locking Increases With Chunk Length

**Figure 2.**
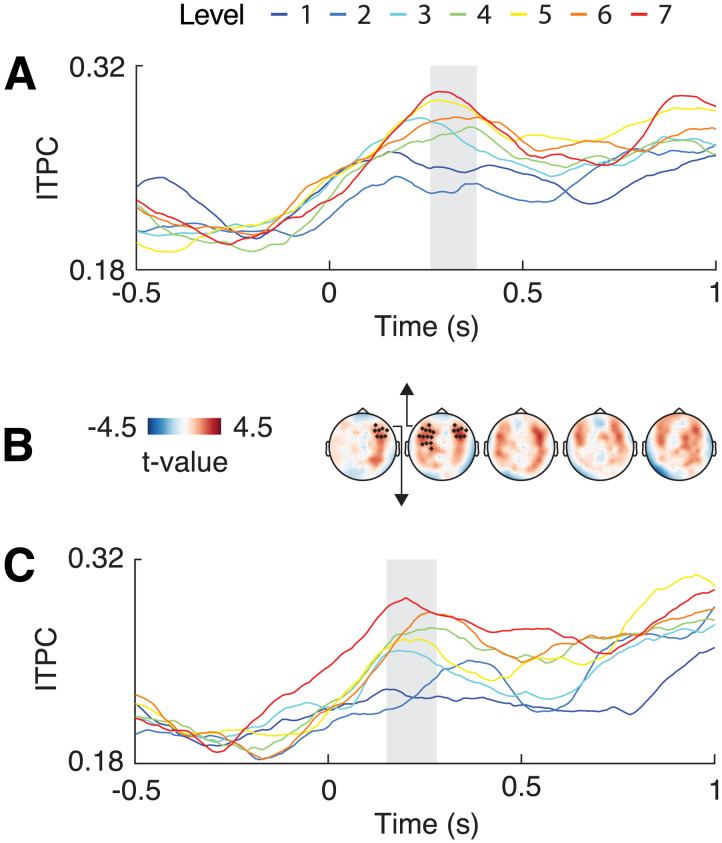
Results of sensor-level inter-trial phase coherence (ITPC) analysis. The analysis was time-locked to the potential boundary position (i.e., the end of the main clause). Cluster-based regression analysis revealed two clusters that showed an increase in ITPC with levels: one left lateralized cluster (A) and one right lateralized cluster (C). Time series are averaged over the magnetometer within each cluster; grey bars indicate the cluster time window. (B) The topography of the *t*-value averaged over segments of 200 ms from 0 to 1 second; filled magnetometers belong to observed clusters.

**Figure 3.**
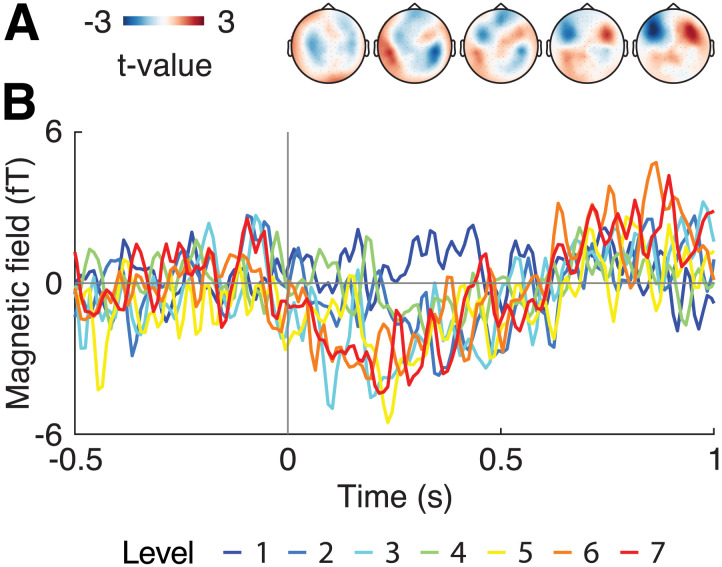
Results of sensor-level ERF analysis, displaying the topography averaged over 200 millisecond segments from 0 to 1 second (A) and time course averaged across all magnetometers (B).

First, we investigated whether phase-locking of low-frequency neural activity (<4 Hz) is related to clause duration: Is the phase angle more consistent across trials when the clause duration aligns with an endogenous oscillatory period? As the end of a processing cycle approaches, the brain may be forced to chunk, resulting in consistency of neural activity, whereas the brain may be more flexible for shorter durations (Henke & Meyer, 2025). A cluster-based linear regression analysis time-locked to the position where a boundary may be inserted (i.e., at the end of the main clause) revealed an increase of ITPC with longer main clause durations in two clusters (left lateralized cluster-sum *t*(39) = 286.83, *p* = 0.012, from 260–380 ms; right lateralized cluster-sum *t*(39) = 267.79, *p* = 0.014, from 150–280 ms; Figure 2). Testing for a boundary-related CPS, the same regression analysis on the broadband ERF showed no statistically significant change in amplitude across levels (−203.78 < all cluster-sum *t*(39) < 162.43, *p* > 0.03; two-sided test; i.e., 0.025 for the positive and negative clusters; Figure 3).

### Increased Phase-Locking Associates With a Larger Amplitude at Assumed Boundaries

**Figure 4.**
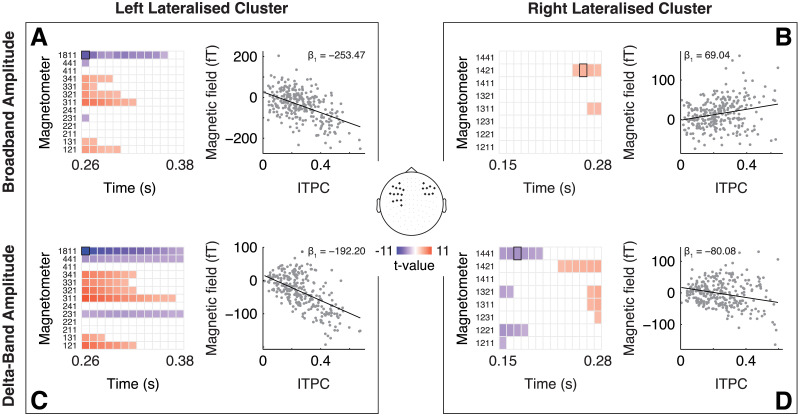
Group-level regression between the ITPC and MEG amplitude. Statistical values of the regression extracted from the magnetometer and timepoints within each cluster of the ITPC and the broadband ERF amplitude (A, B) and the delta-band amplitude (C, D); masked for significant models after Bonferroni-correction (i.e., only significant models are colored). Left panels show the left-lateralized cluster (A, C), and right panels represent the right-lateralized cluster (B, D). Next to the statistical values within the grids, we additionally display a scatterplot of the data with the regression line at the magnetometer and timepoint of the highest absolute *t*-value within each comparison (field outlined in black within each grid).

Next, we tested more specifically whether increased phase-locking within the delta frequency band relates to an increase in amplitude of the ERF at the assumed chunking point. To that end, we extracted the ITPC and amplitude from magnetometers and time points that showed an increase in ITPC across levels. Linear mixed-effects models revealed a relationship between ERF amplitude and ITPC: Within the left-lateralized ITPC cluster, nine magnetometers showed a significant relationship across a time window from 0.26–0.36 (all absolute *t* > 3.74, *p* < 0.04; Bonferroni-corrected; Figure 4A). Within the right-lateralized cluster, we observed two significant magnetometers from 0.25–0.28 s with the broadband ERF amplitude (all *t* > 3.73, *p* < 0.032; Bonferroni-corrected for magnetometers and time points; Figure 4B). Note that opposite to ERPs in the EEG, the sign of the ERF reflects the orientation of the underlying dipole. Therefore, the sign may not be interpreted in the same manner as signs in EEG research. Rather, deviation from zero indicates the magnitude of the magnetic field. For example, in our results, a negative slope may indicate that large ITPC values relate to large negative deviations from zero, while small ITPC values relate to a small magnitude (cf. Figure 4A).

Post hoc, we hypothesized that phase-locking within the delta band should more specifically relate to an increase in ERF amplitude within the same frequency band. To that end, we low-pass filtered the 6-second-long trials at 4 Hz (8^th^-order two-pass Butterworth *IIR* filter, 48dB/octave) and performed the same analysis. The results sharpen the findings on the broadband (0.2–30 Hz) analysis, showing a quantitatively stronger relationship in both the left-lateralized cluster (all absolute *t* > 3.91, *p* < 0.021, Bonferroni-corrected; over the same magnetometers and all timepoints 0.26–0.38 s; Figure 4C) as well as in the right-lateralized cluster (all absolute *t* > 3.69, *p* < 0.042, Bonferroni-corrected; over seven magnetometers between 0.15 and 0.28 s; Figure 4D).

### Phase-Locking Is Generated by Bilateral Temporal and Right Anterior Regions

**Figure 5.**
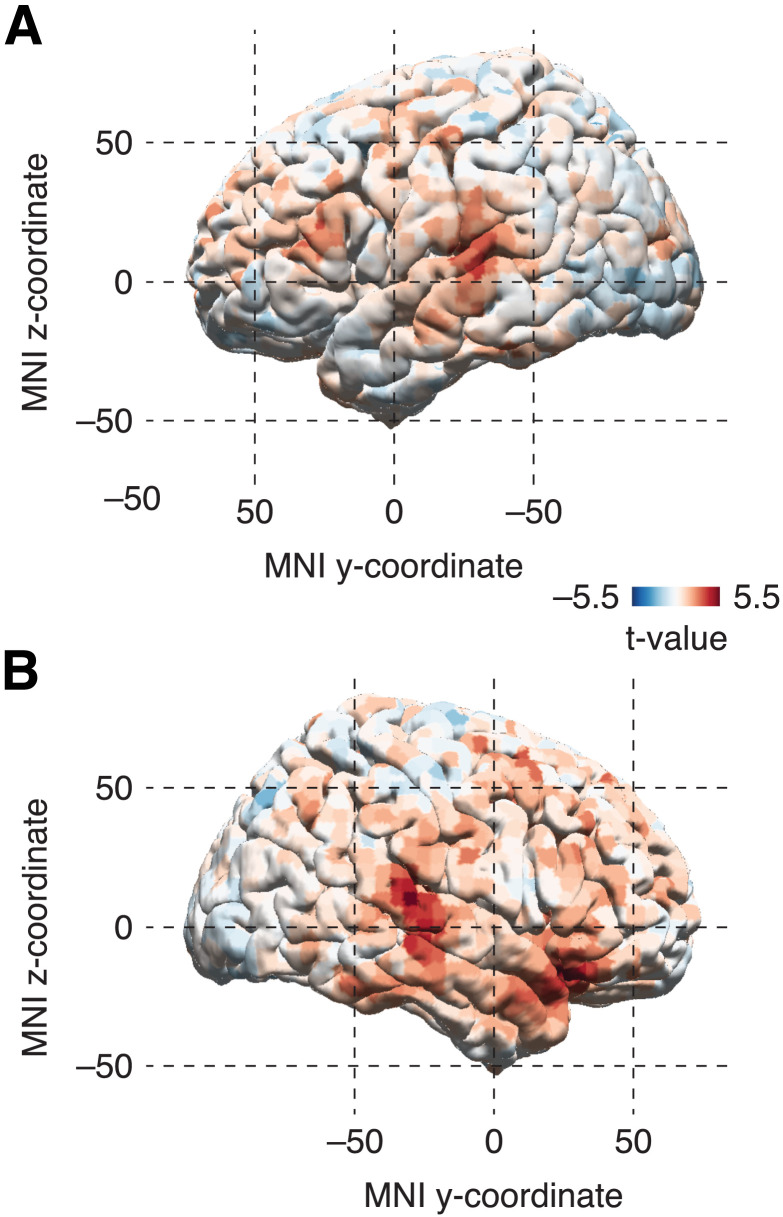
Results of the source-level regression of ITPC across levels; cortical map of *t*-values displayed on the left (A) and right hemisphere (B).

Finally, we aimed to localize the neural source underlying the phase-locking effect. Analogous to the sensor-level analysis, we performed a cluster-based linear regression within atlas-defined cortical source space and observed two clusters showing an increase in delta-band ITPC across levels: One left lateralized cluster in temporal areas (cluster-sum *t*(39) = 287.66, *p* = 0.02; expanding over 29 areas; max *t*-value = 4.24 in the left A5; Figure 5A) and one right lateralized cluster spanning over temporal and frontal regions (cluster-sum *t*(39) = 1224.4, *p* < 0.001; expanding over 73 areas; Figure 5B). Visually, the right-lateralized cluster combines two peaks with one temporal center peaking in the auditory association cortex (area A4; max *t*-value = 5.85) and one more anterior hub expanding over the inferior frontal gyrus (area 47l; max *t*-value = 5.39). Table 2 shows the ten atlas-defined cortical regions with the largest volume within each cluster.

**Table 2.** Source-level results mapped onto atlas-defined cortical regions

**Left lateralized cluster**			**Right lateralized cluster**	
**Area**	**Number of nodes in cluster (percentage)**		**Area**	**Number of nodes in cluster (percentage)**
STSvp	12 / 121 (10%)	**1**	TGd	29 / 486 (6%)
A4	9 / 121 (7%)	**2**	TE1a	20 / 486 (4%)
A5	8 / 121 (7%)	**3**	TE1p	19 / 486 (4%)
STSdp	8 / 121 (7%)	**4**	47s	18 / 486 (4%)
TPOJ1	8 / 121 (7%)	**5**	47l	16 / 486 (3%)
PF	8 / 121 (7%)	**6**	A5	16 / 486 (3%)
TE1p	7 / 121 (6%)	**7**	8Av	15 / 486 (3%)
TE1m	7 / 121 (6%)	**8**	TE2a	14 / 486 (3%)
PFop	6 / 121 (5%)	**9**	STGa	12 / 486 (3%)
STSva	5 / 121 (4%)	**10**	A4	12 / 486 (3%)

*Note*. A4 = auditory 4 complex; A5 = auditory 5 complex; STGa = superior temporal gyrus anterior; STSvp = superior temporal sulcus ventral posterior; STSdp = superior temporal sulcus dorsal posterior; STSva = superior temporal sulcus ventral anterior; PF(op) = parietal area (operculum); TE1(a, m, p) = temporal area 1 (anterior, medial, posterior); TE2a = temporal area 2 anterior; TGd = temporal gyrus dorsal; TPOJ1 = temporo-parieto-occipital-junction 1; 8Av = area 8A ventral; 47(s, l) = area 47 (superior, lateral)

### Behavioral Attachment Is Mediated by Working Memory Span and Phase-Locking

**Figure 6.**
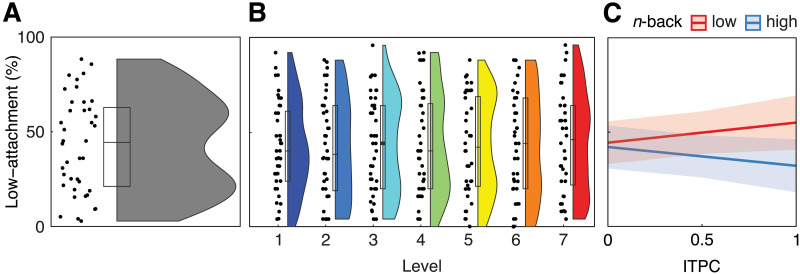
Results of behavioral analysis. After each sentence, participants indicated their attachment via a button press; attachment preferences (A) averaged across levels and (B) across levels. (C) Group-level regression results displaying the predicted attachment in the behavioral task across levels as a function of ITPC divided by memory span group (median split based on *n*-back scores; *N* = 20 per group); shaded areas represent 95% confidence intervals.

After each sentence, participants indicated their attachment decision via a button press. In this way, we could test whether the chunking pattern observed in the neural data is also reflected in the behavioral responses. Single-trial behavioral analysis with a generalized linear mixed effects model indicated no change in attachment preference based on the level (likelihood ratio test against a model without the factor level; *χ*2(6) = 4.43, *p* = 0.6; Figure 6A). Rather, as opposed to our expectations, participants seem to have an attachment preference that remains unchanged across levels; apparently, the behavioral task was not sensitive to the experimental duration manipulation (Figure 6B).

Still, we wanted to test whether the neural results could link to individual attachment preferences. Given previous findings that related working memory to chunking (James et al., 2018; Roll et al., 2012; Schremm et al., 2015; Swets et al., 2007), we also considered working memory group based on a median split of scores in a *n*-back task (Braver et al., 1997; Jaeggi et al., 2010). Median split resulted in two groups of equal size with 20 participants each. We separately extracted ITPC at the timepoint and magnetometer that showed the strongest positive (*t* = 7.45, MEG0311 at 0.26 s) and negative (*t* = −8.96, MEG1811 at 0.26 s) relationship with the evoked amplitude across the broadband data and fitted a separate linear mixed-effects models for each of them. These magnetometers were chosen based on prior work that has found a relation between the amplitude of the CPS and working memory capacity (Schremm et al., 2015). Automatic model selection for the positive relationship revealed a final model for attachment preference, which included a two-way interaction between ITPC and the *n*-back group and random intercepts for participants. The results show a statistically significant interaction of the *n*-back group and ITPC (*t* = 2.27, *p* = 0.02; Figure 6C) without any main effect (both *p* > 0.7). The interaction suggests that low-span subjects show more attachment to NP2 (low attachment in psycholinguistics) with increasing ITPC (estimate = 0.11), while high-span subjects show more attachment to NP1 when ITPC increases (high attachment; estimate = −0.1). As we interpret an increase in ITPC as indicator for the insertion of a boundary, the results of the high-span group are supporting our initial predictions, while the low-span subjects showed a different pattern–possibly suggesting the insertion of a boundary already earlier in the sentence (see Discussion). The automatic model selection for the negative relationship included ITPC and *n*-back group as fixed effects without interaction term and random intercepts for participants. The results indicate no effect of ITPC or *n*-back group on the attachment preference (both *p* > 0.3).

## DISCUSSION

Our results suggest that the brain projects duration-limited integration windows onto language comprehension. Cycles of low-frequency neural activity may be the electrophysiological substrate behind this time constraint. We report that the likelihood of chunking increases with clause duration, as indicated by increased delta-band phase-locking at chunk boundaries. The period of delta-band oscillations may set an endogenous timing constrain on chunking. When sentences are short, participants are variable in their chunking behavior and may or may not insert a chunk boundary. Source localization of the phase-locking effect revealed bilateral temporal areas as well as right anterior areas. Our findings further suggest that a characteristically evoked response at chunk boundaries (i.e., the CPS) might derive from phase-locking of neural oscillations. This supports recent work on naturalistic listening, which has qualitatively related an evoked response at chunk boundaries to speech-tracking at delta-band frequency (Inbar et al., 2023). Lastly, behavioral results indicate inter-individual differences in the integration window, supporting the idea of internally generated processing windows in the brain. Specifically, we show that behavioral sentence comprehension was predicted by an interaction of working memory capacity and delta-band phase-locking.

### Temporal Processing Windows

The current MEG results indicate that forceful prolongation of the integration time window leads to a gradual increase in the likelihood of chunking. This is consistent with the idea that the brain projects temporal processing windows (Hasson et al., 2008; Pöppel, 1997, 2009) onto language comprehension (Hemforth et al., 2015; Jun & Koike, 2008; Lerner et al., 2011). In general, when stimuli necessitate integration across more than 3 s, performance decreases, and neural response patterns change across domains, including sensorimotor integration (Mates et al., 1994), visual (Burr & Santoro, 2001; Gomez et al., 1995; Polgári et al., 2020; van Ee, 2005), and auditory processing (Sams et al., 1993; Wang et al., 2016), as well as time perception (Chen et al., 2015; Kononowicz et al., 2015; Szelag et al., 1996, 2022). Our analyses suggest that inter-individual differences in working memory capacity and a lack of sensitivity of our behavioral task may have blurred the temporal limit on the group level (James et al., 2018; Roll et al., 2012, 2013; Swets et al., 2007). Specifically, the observed interaction between working memory group and delta-band phase-locking suggests that the high-span participants perform in line with our initial hypothesis: inserting a chunk boundary after NP2 as sentences get longer (i.e., when phase-locking increases). In contrast, participants with low working memory capacity may chunk longer sentences already after the NP1, combining the relative clause and the NP2 into one processing window and thus, leading to low-attachment. In other words, the groups may differ in their neural processing window. While individuals with high working memory can integrate information over longer temporal windows, individuals with low working memory span need to chunk earlier.

Notwithstanding the evidence for temporal consistency, the observed variance within and across subjects suggests that the brain mechanism of chunking is still flexible in terms of timing, within limits. This fits with the observed degree of temporal regularity in language, which is substantial, but not perfect (Inbar et al., 2020; Stehwien & Meyer, 2022; Vollrath et al., 1992).

Behavioral sentence comprehension could be predicted by an interaction of working memory capacity and delta-band phase-locking at chunk boundaries. Yet, we did not find a behavioral effect with regard to the duration manipulation. More specifically, participants seem to have an attachment preference (cf. Harding et al., 2019) independent of the duration leading to little variance in individuals’ responses. This makes establishing a direct link to the neural data difficult. Further research is needed to relate chunking-related neural activity to sentence comprehension more directly and to substantiate the functional relevance of chunking. This link will be essential to address alternative interpretations of our results. For instance, besides our interpretation of an endogenous chunking mechanism, the results could also reflect sustained attention or cumulative processing load related to the buildup of information over a longer period of time. We note that those mechanisms would likely elicit differences in the ERF, where we do not observe an effect of duration. Moreover, the observed delta-band phase-locking could also reflect the time-locking of expectations rather than chunking (e.g., Breska & Deouell, 2017). That is, the longer the main clause, the higher the probability for the timepoint where a boundary needs to be inserted. The observed differences in chunking between the working memory groups may speak against this interpretation; however, clearer behavioral results will be needed.

We suggest that the chunking mechanism cuts the language input into multi-word units that fit individual processing limitations. Yet, it remains unclear on which level within the linguistic system this constraint might act. As mentioned above, both the CPS and the delta-band literature records leave it open whether intrinsic timing constraints affect the levels of prosody, implicit prosody, or even syntax. Dissociating (implicit) prosody from syntactic structure (i.e., clauses) is difficult as their boundaries mostly coincide, as would be the case for the present study. While prior work suggests that overt prosody may not be the driving force behind the current and other similar effects (Meyer et al., 2017), future studies are needed to address our rather vague definition of chunking and clarify the operation level of the temporal constraint.

Lastly, our stimuli used synthesized speech. This was the case across all levels. Therefore, we have no reason to believe that our findings—based on the duration manipulation—would differ for natural speech of a human speaker. Future research might extend our findings to natural speech to address this limitation.

### The CPS in EEG Versus MEG

We found delta-band phase to predict MEG amplitude across trials. Yet, unlike prior EEG studies, our analysis did not yield an ERF modulation by experimental level. One likely explanation is that the CPS is invisible to sensor-level MEG: While M– and EEG originate from the same electrophysiological generators, only EEG can see radial generators (Cohen & Cuffin, 1991; Hämäläinen et al., 1993). For music, a boundary-related CPS has been found with MEG (Knösche et al., 2005). Yet, to the best of our knowledge no prior work has reported a CPS at chunk boundaries during language processing in sensor-level MEG. The generators of a sensor-level CPS in EEG have been localized in bilateral auditory cortices, based on MEG reconstruction (Anurova et al., 2022); no CPS was reported on the MEG sensor level. An alternative explanation could be that a CPS has been elicited in all conditions, but the amplitude modulation was not strong enough to reveal differences in the ERF based on the duration manipulation. The here used paradigm does not allow to disentangle those options. Further research is needed to understand the neural source of the CPS and determine whether it can be measured with MEG. Lastly, it could be possible that the amplitude of the CPS might only partially be explained by phase-locked activity; yet possibly related to other mechanisms involved. Therefore, we are cautious in interpreting this finding. Future research may investigate the underlying phase-locking of delta-band oscillations in experimental manipulations where a CPS is actually observed between conditions.

### Neural Mechanism Underlying Chunking

We suggest that cycles of oscillatory activity provide temporal integration windows for multi-word units and might be the mechanism underlying evoked responses at chunk boundaries. However, an alternative explanation could be that the results are driven by evoked responses at the boundaries of the processing windows. For instance, delta-band phase-locking might reflect the processing of chunk onsets (Chalas et al., 2023, 2024) or offsets (Henke et al., 2023) rather than continuous rhythmic activity. To date, there is an ongoing debate in the neural tracking literature, whether the underlying neural mechanism reflects genuine oscillations or merely a series of evoked responses (e.g., Doelling & Assaneo, 2021; Oganian et al., 2023; Zoefel et al., 2018). While we do not observe differences in the evoked responses across levels at the chunk boundaries, we have noted in the previous section that the CPS might be invisible to sensor-level MEG. Therefore, we cannot completely rule out this possibility. Even though the specific neural mechanism remains unclear, both an oscillatory or evoked mechanism would be compatible with the endogenous nature of the time constraint. Specifically, there was no acoustic cue in our experimental manipulation that could elicit a neural response. Thus, any evoked response would still be endogenously driven, and the interpretation of a temporal constraint does not necessarily require an oscillatory mechanism. However, we acknowledge that distinct neural mechanisms have different functional implications and are critical for a mechanistic interpretation (van Bree et al., 2022). Further research will be needed to address the presence of a genuine oscillatory mechanism that triggers the insertion of a chunk boundary when a specific phase angle is reached.

### Neural Structures Underlying the Chunking Mechanism

Source localization revealed bilateral posterior temporal and right anterior sources of the duration-dependent delta-band phase-locking effect. No prior work has analyzed the source of this effect in the context of chunking language (e.g., Henke & Meyer, 2021; Inbar et al., 2023; Meyer et al., 2017). Bilateral auditory areas are generally involved in language comprehension (e.g., Friederici, 2017) and were previously reported for delta-band speech tracking (Bourguignon et al., 2013; Keitel et al., 2017, 2018; Molinaro & Lizarazu, 2018; Park et al., 2015). Furthermore, delta-band tracking of speech in auditory cortices could be explained by prosodic pauses and syntactic processing (Chalas et al., 2024). Additionally, it has been shown that the neural response is enhanced when prosody is aligned with syntactic structure (Degano et al., 2024; Glushko et al., 2022). Therefore, exogenously and endogenously guided mechanisms, both operating at frequencies within the delta band, may interact in order to support successful language processing (Meyer et al., 2020a, 2020b).

Generally, our results suggest a stronger right-lateralized network across fronto-temporal areas. Previous work has associated right auditory cortices with bottom-up processing (e.g., prosody; Bourguignon et al., 2013; Sammler et al., 2015). Specifically, longer timescales involved in suprasegmental auditory processing were suggested to be right-lateralized (Boemio et al., 2005; Giraud et al., 2007; Luo & Poeppel, 2007; Morillon et al., 2012; Oderbolz et al., 2025; Poeppel, 2003; Zatorre et al., 2002). Prosody provides an exogenous cue to chunking (Fletcher, 2010; Frazier et al., 2006; Selkirk, 1984; Steinhauer et al., 1999) and shows rhythmicity within the delta-band frequency (∼1 Hz; Inbar et al., 2020; Stehwien & Meyer, 2022). Accordingly, delta-band activity was prevailing in the right hemisphere (Giroud et al., 2020). The right lateralized source cluster additionally covered areas in the inferior frontal gyrus (IFG), suggested to be involved in parsing the prosodic speech contour (Sammler et al., 2015). Outside of language, the right IFG was found to be involved in the processing of musical structure (Levitin & Menon, 2003; Vuust et al., 2006), possibly suggesting a domain-general mechanism for structuring temporally-evolving stimuli—paralleling the results on the musical CPS (Knösche et al., 2005).

In the present study, phase-locking was not guided by acoustic cues, but based on a duration manipulation that induced chunking from the top down. The functional and temporal overlap between (implicit) prosody and the here discussed temporal integration windows might suggest (partly) overlapping neural mechanisms. One could even speculate that reports of an implicit realization of prosody actually reflect endogenous neural processing windows.

### Conclusion

In summary, delta-band oscillations may be involved in the termination of chunks, possibly reflecting temporal processing windows; when they have reached a specific phase, they may cause chunking as indicated by increased phase-locking. This increase in phase-locking also relates to a commonly observed amplitude increase (i.e., the CPS) at boundaries. Additionally, the here observed overlap between (implicit) prosody and endogenous integration window might indicate shared neural processing mechanisms.

## ACKNOWLEDGMENTS

We thank Laura Riedel and Antonia Schmidt for help with stimulus preparation and Yvonne Wolff-Rosier for data collection.

## FUNDING INFORMATION

Lars Meyer, Max-Planck-Gesellschaft (https://dx.doi.org/10.13039/501100004189), Award ID: MPRG Language Cycles.

## AUTHOR CONTRIBUTIONS

**Lena Henke**: Conceptualization: Equal; Formal analysis: Lead; Investigation: Lead; Methodology: Equal; Writing – original draft: Lead; Writing – review & editing: Equal. **Burkhard Maess**: Investigation: Supporting; Software: Supporting; Writing – review & editing: Supporting. **Lars Meyer**: Conceptualization: Equal; Formal analysis: Supporting; Funding acquisition: Lead; Methodology: Equal; Supervision: Lead; Writing – original draft: Supporting; Writing – review & editing: Equal.

## DATA AVAILABILITY STATEMENT

Raw data cannot be made publicly available due to ethical permissions and legal restrictions. Aggregated data to evaluate the conclusions in the paper are available on Zenodo (https://doi.org/10.5281/zenodo.18742644). Code is available in an OSF repository (https://doi.org/10.17605/OSF.IO/H3C9J).

## Supplementary Material



## TECHNICAL TERMS

Phase-locking: Consistency of phase values.
Narrow-band activity: Neural activity within a limited frequency band.
Implicit prosody: Internal realization of prosodic structure.
Endogenous: Arising or originating internally in the brain.
Speech/Neural tracking: Synchronization between neural activity and exogenous input (e.g., speech).
