# Supplementary material for: How Low-Frequency Neural Activity Structures Language in Time
Source: Neurobiol Lang (Camb). 2026 May 5;7:NOL.a.249. doi: 10.1162/NOL.a.249 (PMC13171203; doi:10.1162/NOL.a.249)
Supplement: Supplementary file 1 [file nol-07-249-s001.pdf]

Supplementary Materials

Stimuli

We created globally ambiguous sentences, where the relative clause could be attached to either of two preceding noun phrases. All sentences followed the same structure (i.e., NP1 *of* NP2 RC) and contained the same number of words. The number of syllables within the words was increased parametrically across 7 levels to create our experimental manipulation (see *Table S1*). For level one and seven, we created 25 sentences. For all other levels, we created 50 sentences. NP-pairs were used twice across different levels, always with different transitive verbs in the main clause as well as a different RC (while the adverb could be the same, the intransitive verb was always different). Generally, intransitive verbs in the RC were only used once within each level, while the adverbs could occur several times. Transitive verbs of the main clause were also repeated twice across levels, except only half of the verbs with one or four syllables were re-used due to the smaller amount of trials in the shortest and longest level. Likewise, the same RC was never repeated with the same transitive verb in the main clause.

Table S1

Detailed overview of the length manipulation based on syllable count.

Main Clause							Relative Clause		
	Name	Transitive verb		NP1		NP2		Adverb	Intransitive verb
	1	1		2		2			
	2	2		2		2			
	2	2		3		3			
Mr. / Ms.	3	3	the	3	of the	3	who	1/2	2/3
	3	3		4		4			
	4	4		4		4			
	4	4		5		5			
